# Resistance mechanisms to inhibitors of p53-MDM2 interactions in cancer therapy: can we overcome them?

**DOI:** 10.1186/s11658-021-00293-6

**Published:** 2021-12-15

**Authors:** Lucia Haronikova, Ondrej Bonczek, Pavlina Zatloukalova, Filip Kokas-Zavadil, Martina Kucerikova, Philip J. Coates, Robin Fahraeus, Borivoj Vojtesek

**Affiliations:** 1 RECAMO, Masaryk Memorial Cancer Institute, Zluty kopec 7, 656 53 Brno, Czech Republic; 2 Department of Medical Biosciences, Umea University, 901 87 Umea, Vasterbotten, Sweden; 3 National Centre for Biomolecular Research, Faculty of Science, Masaryk University, Kamenice 5, 625 00 Brno, Czech Republic; 4 Inserm UMRS1131, Institut de Génétique Moléculaire, Université Paris 7, Hôpital St. Louis, 75010 Paris, France

**Keywords:** p53, MDM2, MDM2 inhibitor, Nutlin-3a, Resistance, Combination therapy, Personalised medicine

## Abstract

Since the discovery of the first MDM2 inhibitors, we have gained deeper insights into the cellular roles of MDM2 and p53. In this review, we focus on MDM2 inhibitors that bind to the p53-binding domain of MDM2 and aim to disrupt the binding of MDM2 to p53. We describe the basic mechanism of action of these MDM2 inhibitors, such as nutlin-3a, summarise the determinants of sensitivity to MDM2 inhibition from p53-dependent and p53-independent points of view and discuss the problems with innate and acquired resistance to MDM2 inhibition. Despite progress in MDM2 inhibitor design and ongoing clinical trials, their broad use in cancer treatment is not fulfilling expectations in heterogenous human cancers. We assess the MDM2 inhibitor types in clinical trials and provide an overview of possible sources of resistance to MDM2 inhibition, underlining the need for patient stratification based on these aspects to gain better clinical responses, including the use of combination therapies for personalised medicine.

## Introduction

p53, the guardian of the genome, has been known for more than 40 years. Its importance as a tumour suppressor has been described from many points of view. In response to cellular stress stimuli, p53 acts as a transcriptional regulator of target genes in growth arrest/senescence and DNA damage repair, interacts with mitochondrial proteins involved in apoptosis, induces the immune response, and has many more roles [1–3]. In normal conditions, the p53 protein level is kept low by its main negative regulator, MDM2 (mouse double minute 2 homologue), which promotes p53 ubiquitination and its subsequent degradation [4, 5]. After stress stimuli, the MDM2-p53 interaction is disrupted and p53 increases rapidly to activate p53 responses [6]. In a negative feedback loop, p53 transcriptionally upregulates MDM2 levels [7, 8]. The importance of the MDM2-p53 interaction is underlined by transgenic mice, where *Mdm2*-null mice show embryonic lethality due to massive apoptosis, which is rescued by concomitant *Trp53* deletion [9, 10].

*TP53* is the most commonly mutated gene in human cancer. Although p53 retains its wild-type form in around 50% of cancers, its function is compromised by other means in most of these tumours [11, 12]. Overexpression of MDM2 by gene amplification or single nucleotide polymorphism is documented in many cancer types, and the occurrence of p53 mutations and overexpression of MDM2 are usually mutually exclusive, supporting the notion that MDM2 overexpression is responsible for driving the cancer phenotype by abolishing p53 activity [13–15]. Other MDM2 functions may also contribute to its oncogenic effects, such as its pro-angiogenic activity, involvement in chromosome instability, degradation of cell cycle regulators, and degradation of E-cadherin leading to epithelial–mesenchymal transition (EMT) [16–22]. New findings also suggest that MDM2 overexpression confers resistance to conventional chemotherapy [23]. The use of compounds that disrupt the p53-MDM2 interaction is therefore a rational approach to activate the p53 response in cancer cells in which p53 activity is compromised by mechanisms other than *TP53* mutation.

Initial structural characterisation of the p53-MDM2 binding interface revealed that the MDM2 N-terminus possesses a deep hydrophobic pocket occupied by side chains of three amino-acid residues (Phe19, Trp23, Leu26) in the alpha-helical transactivation domain of p53 [24]. The development of small molecule inhibitors and stapled peptides that bind this pocket is mainly based on mimicking these three amino acid side chains and later inhibitors provide one additional binding site to achieve higher affinity [25–27]. This approach was shown to inhibit the p53-MDM2 interaction and to activate p53 responses. Nutlins were the first class of small inhibitor molecules [28] and a racemic mixture was used initially (referred to as nutlin-3 in the text). Subsequently, an active enantiomer called nutlin-3a became more widely used (see MDM2 inhibitor types and clinical trials for more details). From a structural point of view, MDM2 displays high plasticity, and the binding of p53 and some small molecule inhibitors induce ordering of the MDM2 N-terminal domain [29–32]. A second responsive site was identified in the N-terminal domain for nutlin-3 [33]. Importantly, inhibitors that bind the MDM2 N-terminal domain do not disturb the ubiquitination activity of the MDM2 RING domain present in the C-terminus [34].

In this review, we focus on MDM2 inhibitors that were designed to disrupt MDM2-p53 binding and thus activate wild-type p53, such as nutlin. Many small molecule inhibitors have entered clinical trials, often in combination with other therapeutics. Patients were originally stratified for MDM2 inhibitor treatment based on their p53 status or MDM2 amplification [35]. Although activation of wild-type p53 is almost universal after MDM2 inhibition, the outcomes range from cell cycle arrest to apoptosis, depending on cell type, dose and time of exposure [36–41]. Therefore, to stratify patients who will benefit from MDM2 inhibition, it is necessary to identify criteria other than simple p53 wild-type/mutation status that govern the cellular response to such treatment. The precise characterisation of tumour genetic background and cancer type should improve the response to MDM2 inhibition and could help to design appropriate combination therapies. Improved schedules and doses of treatment combinations should also help to mitigate problems with toxicity and/or acquired resistance.

## Types of MDM2 inhibitors and clinical trials

**Fig. 1:**
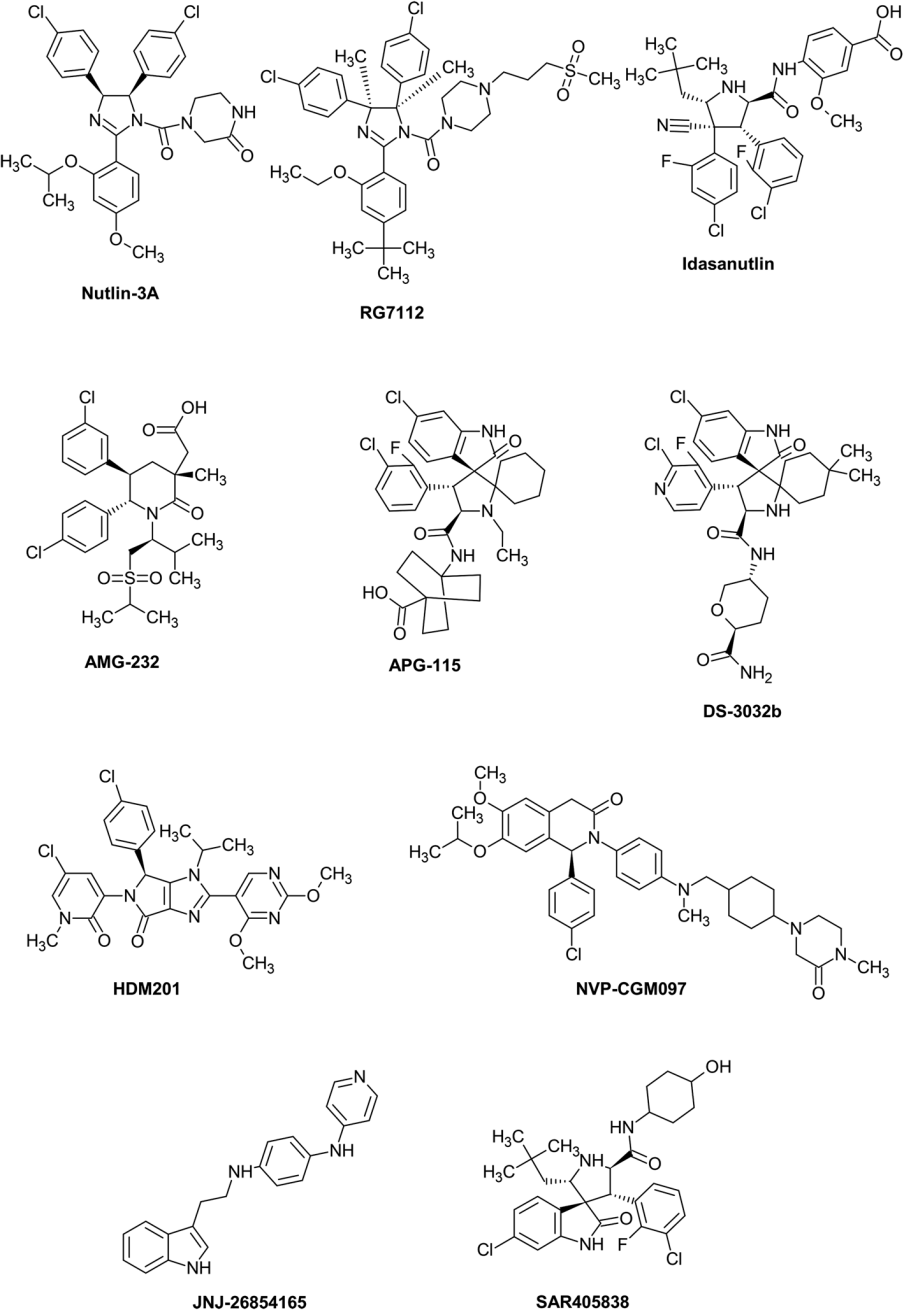
Structures of MDM2 inhibitors. Available structures were obtained from PubChem database [42] with accession numbers CID 11433190; CID 57406853; CID 53358942; CID 58573469; CID 91972012; CID 89051543; CID 71678098; CID 53240420; CID 11609586; CID 53476877 and plotted by ACD/ChemSketch, version 2021.1.1

The first small molecules that inhibit MDM2/p53 binding were synthesised by Vassilev et al. [28] as racemic mixtures of compounds called **nutlin** 1–3, from which the most potent binding was reported by enantiomer 3a (IC_50_ ~ 90 nM) (Fig. 1). All these early nutlins are *cis*-imidazoline analogues that mimic the natural helical order of peptides, and three side groups of imidazoline scaffold exactly fit into the MDM2 groove that binds to p53. Although early nutlins showed cellular activity and confirmed the concept of MDM2 inhibition to activate wild-type p53, they lack the required pharmacological properties for clinical development and trials.

The first clinically tested molecule was **RG7112** (IC_50_ ~ 20 nM). Compared to nutlin-3a, it differs in substitution of the imidazoline core and replacement of the methoxy group by a *tert*-butyl group [43]. RG7112 was the first MDM2 inhibitor clinically assessed in a trial registered with EudraCT (2009-015522-10) in patients with MDM2-amplified liposarcomas [35]. Clinical activity as monotherapy or in combination with cytotoxic drugs such as cytarabine or doxorubicin was also assessed in patients with solid tumours, haematological neoplasms or sarcomas in several phase I and Ib clinical trials (Table 1). A clinical response was achieved particularly against AML (NCT00623870), even in cases carrying p53 mutations [44]. However, RG7112 showed poor tolerability at the required high doses, with relatively severe haematological and gastrointestinal toxicities that hampered achieving appropriate clinical effects, and RG7112 is not currently under clinical assessment.

**Table 1:** List of MDM2-p53 inhibitors in completed clinical trials

Drug	Disease	Combination with	Action	Phase	Status	Trial nr	Sponzor	Time
RG7112 (RO5045337)	Advanced solid tumors			I	Completed	NCT00559533*	Hoffmann-LaRoche	2007–2012
	Hematologic neoplasm			I	Completed	NCT00623870*	Hoffmann-La Roche	2008–2012
	Solid tumors			I	Completed	NCT01164033*	Hoffmann-La Roche	2010–2013
	Sarcoma	Doxorubicin	DNA damage	Ib	Completed	NCT01605526*	Hoffmann-La Roche	2012–2013
	Acute myelogenous leukemia (AML)	Cytarabine	DNA damage	Ib	Completed	NCT01635296*	Hoffmann-La Roche	2012–2013
	Extension study of studies marked with*			I	Completed	NCT01677780	Hoffmann-La Roche	2012–2017
Idasanutlin (RG7388)	Advanced malignancies, except leukemia			I	Completed	NCT01462175	Hoffmann-La Roche	2011–2014
	Solid tumors			I	Completed	NCT03362723	Hoffmann-La Roche	2017–2019
	Acute myelogenous leukemia	Idarubicin Daunorubicin Cytarabine	DNA damage DNA damage DNA damage	I/Ib	Completed	NCT01773408	Hoffmann-La Roche	2013–2016
	Relapsed and refractory AML	Cytarabine	DNA damage	III	Terminated	NCT02545283	Hoffmann-La Roche	2012–2020
	Non-Hodgkin’s lymphoma	Obinutuzumab Rituximab	Anti-CD20 Anti-CD20	I/Ib	Terminated	NCT02624986	Hoffmann-La Roche	2015–2019
	Relapsed and refractory AML	Venetoclax	BCL-2 inhibitor	Ib	Completed	NCT02670044	Hoffmann-La Roche	2016–2020
	Relapsed and refractory follicular lymphoma, relapsed and refractory diffuse large B-cell lymphoma	Obinutuzumab Venetoclax Rituximab	Anti-CD20 BCL-2 inhibitor Anti-CD20	Ib/II	Terminated	NCT03135262	Hoffmann-La Roche	2018–2020
	Acute myelogenous leukemia	Cytarabine Daunorubicin	DNA damage DNA damage	Ib/II	Completed	NCT03850535	Hoffmann-La Roche	2019–2020
AMG-232 (KRT-232)	Advanced solid tumors, multiple myeloma			I	Completed	NCT01723020	Amgen	2012–2017
	Acute myelogenous leukemia	Trametinib	MEK inhibitor	I	Completed	NCT02016729	Kartos Therapeutics, Inc.	2014–2017
	Metastatic melanoma	Trametinib Dabrafenib	MEK inhibitor BRAF inhibitor	Ib/IIa	Completed	NCT02110355	Kartos Therapeutics, Inc.	2014–2018
APG-115 (AA-115)	Advanced solid tumors. Lymphomas			I	Completed	NCT02935907	Ascentage Pharma Group, Inc.	2016–2019
CGM097	Advanced solid tumors with TP53wt			I	Completed	NCT01760525	Novartis Pharmaceuticals	2013–2019
HDM201	Liposarcoma	Ribociclib	CDKinhibitor	Ib/II	Completed	NCT02343172	Novartis Pharmaceuticals	2015–2019
DS-3032b (Milademetan)	Advanced solid tumors, lymphomas			I	Completed	NCT01877382	Daiichi Sankyo Co., Ltd.	2013–2020
	Relapsed and refractory AML			I	Completed	NCT03671564	Daiichi Sankyo Co., Ltd.	2018–2019
	Acute myelogenous leukemia	Quizartinib	Tyrosine kinase inhibitor	I	Terminated	NCT03552029	Daiichi Sankyo Co., Ltd.	2018–2021
	Acute myelogenous leukemia, myelodysplastic syndromes	5-Azacitidine	DNA damage	I	Terminated	NCT02319369	Daiichi Sankyo Co., Ltd.	2014–2021
ALRN-6924	Advanced solid tumors, lymphomas			I/IIa	Completed	NCT02264613	Aileron Therapeutics	2014–2020
	Acute myelogenous leukemia, myelodysplastic syndromes	Cytarabine	DNA damage	I/Ib	Completed	NCT02909972	Aileron Therapeutics	2016–2019
JNJ-26854165	Advanced of refractory solid tumors				Completed	NCT00676910	Johnson & Johnson Pharmaceutical Research & Development, L.L.C.	2006–2010
SAR405838	Solid tumors	Pimasertib	MEK inhibitor	I	Completed	NCT01985191	Sanofi	2013–2016

*These studies were extended by clinical trial NCT01677780

Further research left the imidazoline backbone and focussed on pyrrolidine derivatives, which made it possible to improve biological parameters and reduce the effective dose. Idasanutlin (**RG7388**, RO5503781; IC_50_ = 6 nM) is a potent and selective candidate with a better pharmacokinetic profile than RG7112 [45]. Patient responses were evaluated in monotherapy or in combination with chemotherapeutic agents, or with venetoclax (BCL2 inhibitor), posaconazole (CYP3A4 (cytochrome P450 3A4)) inhibitor) or cobimetinib (MEK inhibitor) in haematological malignancies (Table 1). A study using idasanutlin in combination with cytarabine (NCT01773408) showed good tolerability and concluded that MDM2 protein is a predictive biomarker to identify patients who might benefit from idasanutlin-based therapy [46]. The combined effect of idasanutlin and cytarabine was clinically assessed in a global phase III study (NCT02545283) in patients with AML, but the study was terminated for futility based on efficacy results. Clinical trials evaluating idasanutlin in combination with other agents are summarised in Table 1. Several other idasanutlin-based trials are currently in progress (Table 2).

**Table 2:** List of MDM2-p53 inhibitors in ongoing clinical trials

Drug	Disease	Combination with		Phase	Status	Trial nr	Sponzor	Start date
Idasanutlin (RG7388)	Breast cancer	Atezolizumab	Anti-PD-L1	I/II	Active, not recruiting	NCT03566485	Vanderbilt-Ingram Cancer Center	2018
	Acute myelogenous leukemia (AML), acute lymphocytic leukemia, neuroblastoma, solid tumors	Cyclophosphamide Topotecan Fludarabine Cytarabine		I/II	Recruiting	NCT04029688	Hoffmann-La Roche	2020
	Relapsed multiple myeloma	Ixazomib Dexamethasone Venetoclax		I/II	Active, not recruiting	NCT02633059	Mayo Clinic	2021
AMG-232 (KRT-232)	Acute myelogenous leukemia, relapsed and refractory AML	Decitabine	DNA damage	I	Recruiting	NCT03041688	National Cancer Institute	2017
	Soft tissue sarcoma	Radiation therapy		Ib	Recruiting	NCT03217266	National Cancer Institute	2017
	Polycythemia vera	Ruxolitinib	TK inhibitor	II	Active, not recruiting	NCT03669965	Kartos Therapeutics, Inc	2018
	Relapsed multiple myeloma	Carfilzomib Dexamethasone Lenalidomide	Proteosome inhibitor Chemotherapy Chemotherapy	I	Recruiting	NCT03031730	National Cancer Institute	2017
	Brain cancer	Radiation therapy		I	Recruiting	NCT03107780	National Cancer Institute	2018
	Acute myelogenous leukemia	Cytarabine Idarubicin HCl	DNA damage DNA damage	Ib	Recruiting	NCT04190550	National Cancer Institute	2020
APG-115 (AA-115)	Metastatic melanomas, advanced solid tumors	Pembrolizumab	Anti-PD-1	Ib/II	Recruiting	NCT03611868	Ascentage Pharma Group, Inc.	2018
	Salivary gland carcinoma	Carboplatin	DNA damage	I/II	Recruiting	NCT03781986	Ascentage Pharma Group, Inc.	2019
	Acute myelogenous leukemia (AML), acute lymphocytic leukemia, neuroblastoma	Azacitidine Cytarabine	DNA damage DNA damage	Ib	Recruiting	NCT04275518	Ascentage Pharma Group, Inc.	2020
	Acute myelogenous leukemia	5-azacitidine	DNA damage	Ib/II	Recruiting	NCT04358393	Ascentage Pharma Group, Inc.	2020
	Liposarcoma, advanced solid tumors	Toripalimab	Anti-PD-1	Ib/II	Not yet recruiting	NCT04785196	Ascentage Pharma Group, Inc.	2021
	T-prolymphocytic leukemia	APG-2575		IIa	Not yet recruiting	NCT04496349	Ascentage Pharma Group, Inc.	2021
BI907828	Solid tumors			Ia/Ib	Recruiting	NCT03449381	Boehringer Ingelheim	2018
	Solid tumors	Ezanbenlimab BI754111	Anti-PD-1 Anti-LAG-3	Ia/Ib	Recruiting	NCT03964233	Boehringer Ingelheim	2019
HDM201 (Siremadlin)	Uveal melanoma	LXS196	PKC inhibitor	I	Recruiting	NCT02601378	Novartis Pharmaceuticals	2016
	Advanced/metastatic colorectal cancer	Trametinib	MEK inhibitor	I	Recruiting	NCT03714958	Centre Leon Berard	2018
	Myelofibrosis	Ruxolitinib	TK inhibitor	I/II	Recruiting	NCT04097821	Novartis Pharmaceuticals	2019
	Range of cancers	Spartalizumab	Anti-PD-1	I	Recruiting	NCT02890069	Novartis Pharmaceuticals	2016
	Malignant solid tumors	Ribociclib	CDK inhibitor	II	Recruiting	NCT04116541	Centre Leon Berard	2020
	Acute myelogenous leukemia	Midostaurin	TK inhibitor	I	Recruiting	NCT04496999	University Hospital Inselspital, Berne	2020
	Acute myelogenous leukemia, myelodysplastic syndromes	MBG453 (Sabatolimab) Venetoclax	Anti-Tim3 BCL-2 inhibitor	Ib	Recruiting	NCT03940352	Novartis Pharmaceuticals	2021
DS-3032b (Milademetan)	Acute myelogenous leukemia, relapsed and refractory AML	Cytarabine Venetoclax	DNA damage BCL-2 inhibitor	I/II	Recruiting	NCT03634228	M.D. Anderson Cancer Center	2018
ALRN-6924	Pediatric cancer	Cytarabine	DNA damage	I	Recruiting	NCT03654716	Dana-Farber Cancer Institute	2018
	Small cell lung cancer	Topotecan		Ib/II	Recruiting	NCT04022876	Aileron Therapeutics	2019
	Breast cancer, malignant solid neoplasm	Paclitaxel		Ib	Recruiting	NCT03725436	M.D. Anderson Cancer Center	2019

*TK* tyrosine kinase, *PKC* protein kinase C

A de novo design of a piperidinone scaffold and addition of an *N*-alkyl substituent led to AM-8553, a predecessor of **AMG-232**. The final structure of AMG-232 is a sulfone piperidinone derivative with two isopropyl groups on the sulfone side chain, and has excellent pharmacokinetic properties (IC_50_ = 10 nM) [47, 48]. AMG-232 showed clinical activity as monotherapy [49] and in combination with trametinib (MEK inhibitor) [50] and/or the BRAF inhibitor dabrafenib [51] (Table 1). Several trials assessing AMG-232 in combination with various agents and radiation therapy are currently recruiting (Table 2).

Promising prospects were given to spirooxindole-containing MDM2 inhibitors **AA-115/APG-115**, with good chemical stability and excellent oral pharmacokinetics. Following oral administration, tumour regression has been observed in xenograft models of acute leukaemia and other cancers, leading to its entry into clinical development [52, 53]. To date, only one study (NCT02935907) assessing APG-115 as monotherapy in patients with advanced solid tumours has been completed [54]; APG-115 was well tolerated, had manageable adverse events and the maximum tolerated dose was recommended for phase II. Preliminary results from a phase II study in combination with pembrolizumab (PD-1 blockade) seem promising for patients with metastatic melanoma or advanced solid tumours resistant to previous immuno-oncologic treatment (NCT03611868) [55]. Other clinical trials of APG-115 are ongoing.

From Boehringer Ingelheim came a compound with a multi-cyclic core called **BI-907828**. The first pharmacokinetic trials across species showed high permeability, good physiological solubility and low systemic clearance together with a promising low human efficacious dose [56]. BI-907828 showed significant anti-tumour activity for patient-derived xenografts from dedifferentiated liposarcomas [57] and patients are recruited to two clinical trials in combination therapy.

**NVP-CGM097** (Novartis) is a representative small molecule with a dihydroisoquinolinone scaffold about four times more potent than nutlin-3a [58, 59]. To date only a phase I dose escalation study in patients with advanced solid tumours has been completed (NCT01750525). Despite haematologic toxicity with delayed-onset thrombocytopenia frequently observed, the tolerability of NVP-CGM097 appeared manageable and the disease control rate was 39% [60]. At the moment there are no planned studies reported.

Another candidate from Novartis, **siremadlin** (**NVP-HDM201**), is an imidazolopyrrolidinone analogue, and experimental data on xenografts showed up to tenfold potentiation compared to NVP-CGM097 [61]. The first data of combined treatment with midostaurin of AML cells harbouring FLT3-ITD (Fms related receptor tyrosine kinase 3 internal tandem duplication) look promising [62]. In clinical trials, NVP-HDM201 showed promising anti-leukaemic activity [63] in patients with wild-type *TP53* (NCT02143635), and clinical safety and efficacy in combination with LEE011 (CDK4/6 inhibitor) were confirmed in patients with liposarcoma (NCT02343172) [64]. Additional clinical studies using NVP-HDM201 are ongoing.

The dispiropyrrolidine based compound **milademetan** (**DS-3032b**), demonstrated in vitro and in vivo reactivation of p53 signalling in neuroblastoma cells, reducing proliferative capacity and causing cytotoxicity [65]. Clinically, DS-3032b as a single agent had an acceptable safety profile and clinical benefit was seen in patients with advanced solid tumours or lymphomas with aberrant MDM2 signalling and wild-type p53 (NCT01877382) [66]. Patients are recruiting to one other study.

Another class of MDM2 inhibitors is cell penetrating stapled alpha-helical peptides designed to bind to both MDM2 and MDMX in nanomolar affinities to disrupt their interaction with p53. The most promising appear to be **ATSP-7041** and its analogue **ALRN-6924** (Aileron Therapeutics) [67, 68]. ALRN-6924 markedly improves survival in AML xenograft models [69]. Clinically, ALRN-6924 was evaluated as monotherapy and in combination with cytarabine in patients with haematologic neoplasms (NCT02909972) and has advanced into a phase I/II clinical study in patients with advanced solid tumours or lymphomas retaining wild-type p53 (NCT02264613). ALRN-6924 was well tolerated and the most frequent adverse side-effects were gastrointestinal [68]. Three additional clinical trials of ALRN-692 are ongoing.

Additionally, other types of MDM2 inhibitors have been developed, including those that block its E3 ligase activity such as HLI98 [70], **JNJ-26854165** [71], **MEL23** and **MEL24** [72], that block heterodimerisation between MDMX and MDM2 such as **MMRi6** and its analogue **MMRi64** [73], and that block the RNA-binding activity of MDM2 [74]. These have not entered clinical trials so far.

## Sensitivity to MDM2 inhibitors

Although several approaches to inhibit MDM2 function have been and are being developed, as outlined above, our review concentrates particularly on the most intensively investigated class of such inhibitors, those that target the binding interface of MDM2 to p53, such as nutlin. Despite their apparent uniformity and simplicity of action, a broad spectrum of responses to such agents is documented, implying that the overall outcome of p53 activation after MDM2 inhibition is influenced by upstream and downstream p53 signalling pathways. Indeed, numerous factors have now been shown to influence the response to MDM2 inhibition, demonstrating the need to understand the complex mechanism(s) involved if these agents are to fulfil their clinical promise. Here, we discuss factors affecting MDM2 inhibition efficacy and their potential for patient stratification.

### p53-dependent determinants

**Fig. 2:**
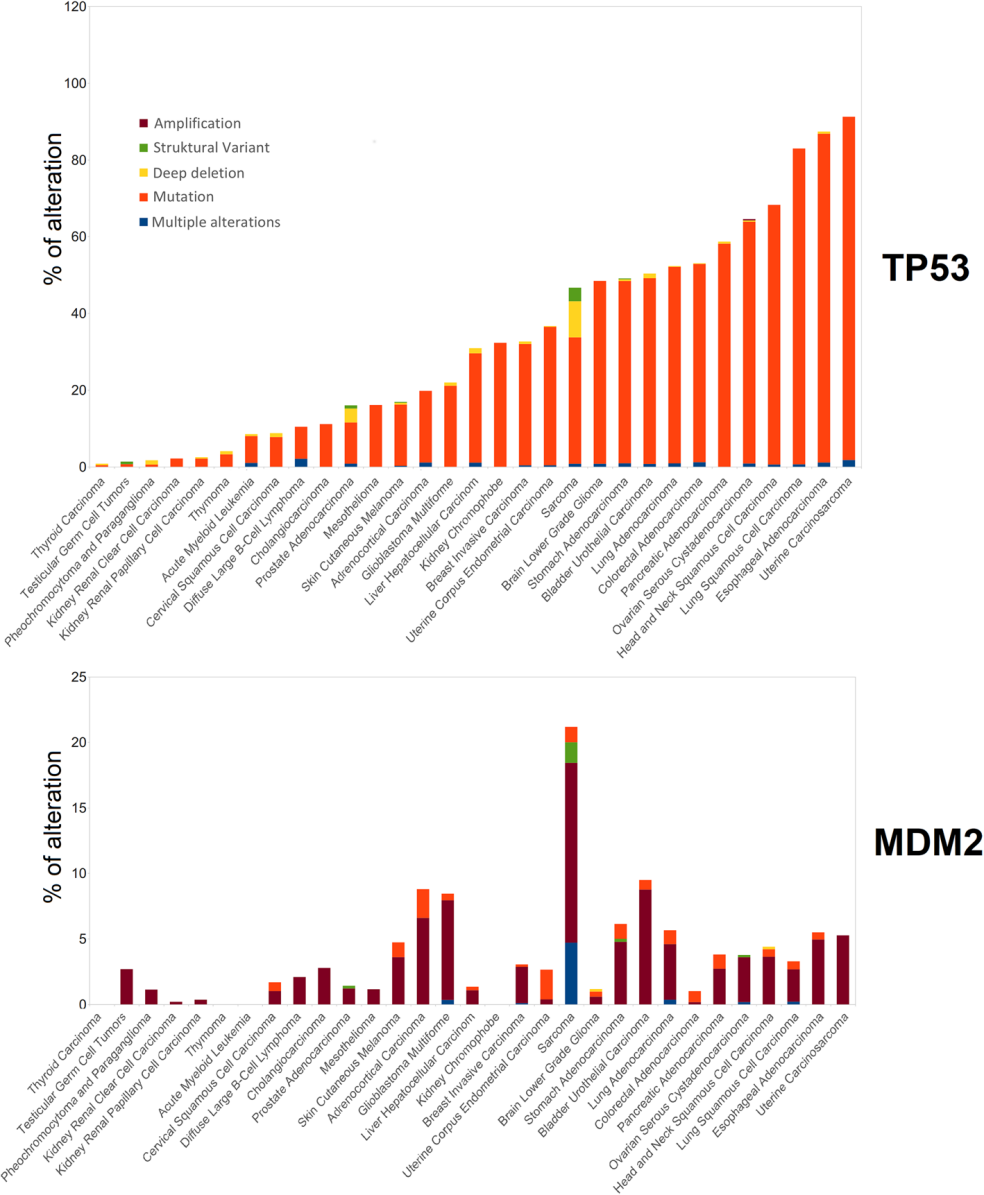
Cancer-associated alteration in *TP53* and *MDM2.* The distribution of alterations in *TP53* and *MDM2* genes divided by cancer type (colour coding: deep red = amplification, green = structural variation, yellow = deep deletion, red = mutation, dark blue = multiple alterations). The cancer types are ordered from lowest to highest percent of *TP53* alterations. The data were obtained from TCGA [83]

**Fig. 3:**
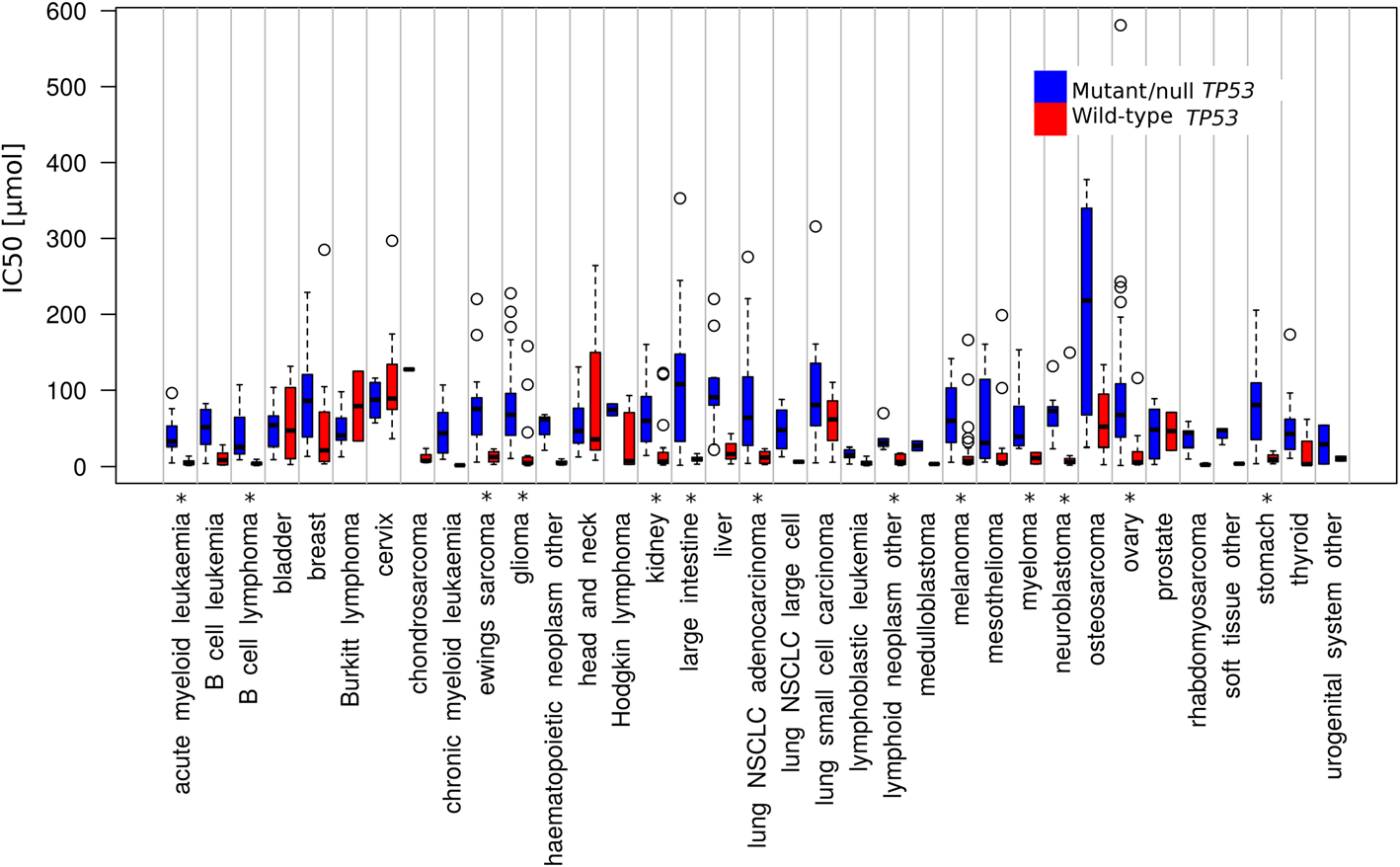
IC_50_ values for nutlin-3a across cancer types. Box-plot of IC_50_ values for human cancer cell lines divided by tissue subtype. Cell lines from each tissue subtypes are divided based on their *TP53* status into wild type (red) and mutant/null (blue) groups. Differences in IC_50_ values between the two groups were evaluated by Mann–Whitney test; *p < 0.01. Data were obtained from Genomics of Drug Sensitivity in Cancer (dataset GDSC1; GDSC; [78]), and the IARC TP53 database (version R20, July 2019; [79]) was used for distribution according to p53 status

Generally, p53 status is the major determinant of response [36, 40, 75, 76]. The use of MDM2 inhibitors in cancer types with low p53 mutation frequencies, such as thyroid carcinoma, acute myeloid leukaemia (AML), melanoma and others (Fig. 2), gave hope for this approach to be widely used in cancer treatment. The first experiments demonstrated that MDM2 inhibitors cause cell cycle arrest and apoptosis in wild-type p53 cancer cells, whereas only transient cell cycle arrest and minimal accumulation of p53 with low cytotoxic effects were observed in normal cells in animal models [39, 77]. However, a wider panel of p53 wild-type cells indicated that the response to MDM2 inhibitors ranges from cell cycle arrest to apoptosis [40]. We analysed *TP53* status in relation to nutlin-3a sensitivity in 947 cell lines using data from the Genomics of Drug Sensitivity in Cancer database (GDSC1) [78]. We divided cell lines into those with wild-type or mutant/null p53 using data from the IARC TP53 database (version R20, July 2019 [79]). Figure 3 shows the distribution of nutlin-3a IC_50_ values in these two groups, with a clear dependence on *TP53* status for most tissue subtypes. However, only around half of the tissue subtypes show statistical significance and the distribution of IC_50_ values is wide in some cases, seen for example for bladder, breast and osteosarcoma, indicating that criteria other than p53 mutation also influence nutlin-3a sensitivity. Moreover, we should consider *TP53* mutations in a broader perspective, since not all *TP53* mutations have the same impact on p53 inactivation [80]. For example, although many p53 mutations occur in the DNA binding domain and affect transactivation, other mutations impact protein–protein interactions [81]. Moreover, some mutations induce p53 gain of function, giving the tumour additional growth/survival advantages [82], and activating p53 in these cases may have an opposite effect.

#### MDM2 status

Apart from *TP53* mutations, **MDM2** overexpression is a common means by which the p53 pathway is inactivated. *MDM2* gene amplification is found in various tumours, most notably sarcomas (Fig. 2), and tends to occur mutually exclusively with *TP53* mutation. A single nucleotide polymorphism in the *MDM2* promoter (SNP309) and deletion of the *ARF-INK4a* locus are other means for MDM2 overexpression [14, 84]. Thus, MDM2 inhibition is potentially promising especially for MDM2 amplified tumours with wild-type p53, and MDM2 protein level is a determinant of the response to MDM2 inhibitor treatment in vitro [40, 85, 86]. However, no correlation was observed between MDM2 amplification and effectiveness of response in AML [87]. Moreover, no correlation was observed between MDM2 and apoptotic outcome of nutlin-3 in sarcomas [88], and a clinical trial of patients with MDM2-amplified liposarcomas yielded a poor response to MDM2 inhibition as monotherapy [35], suggesting that *MDM2* amplification is not a universal marker for therapy response. Again, the simplistic notions of using MDM2 inhibition in cancer therapy are not borne out in the clinic, despite evidence in their favour from in vitro models.

#### MDMX status

Indeed, several mechanisms interfering with MDM2 inhibition have been described. One of the first came from the MDM2 family member **MDMX**. Like MDM2, MDMX overexpression is common in several tumour types and represents an alternative mechanism of p53 inactivation [89–91]. Although MDMX and MDM2 share the same binding site on p53, there are differences in their binding mode [92], and several MDM2 inhibitors do not bind efficiently to MDMX [47, 93, 94]. However, MDMX forms a complex with MDM2 and enhances its ability to ubiquitinate p53, MDM2 and MDMX itself [95–97]. Consequently, the ratio between MDM2 and MDMX levels plays a critical role in p53 regulation [96]. The complexity of these interactions is underlined by evidence that MDM2 and MDMX binding can be enhanced by p53 and nutlin-3a [98]. Furthermore, nutlin-3a was ineffective in tumours overexpressing MDMX, and this resistance was reversed following deletion of the MDMX RING domain that is important for the interaction with MDM2 [99, 100]. In line with this, a search for factors that interfere with MDM2 inhibition identified MDMX as one of the main drivers of resistance [101], and MDMX overexpression correlates with poor response to nutlin-3a in chronic lymphocytic leukaemia (CLL) cells [102] but not in AML cells [87]. Due to the involvement in MDM2 inhibition resistance and because MDMX is itself involved in cancer development, MDMX became a target of anti-tumour treatment and several MDMX or dual MDM2-MDMX inhibitors were developed (summarised in Types of MDM2 inhibitors).

#### Cell cycle influences

**Fig. 4:**
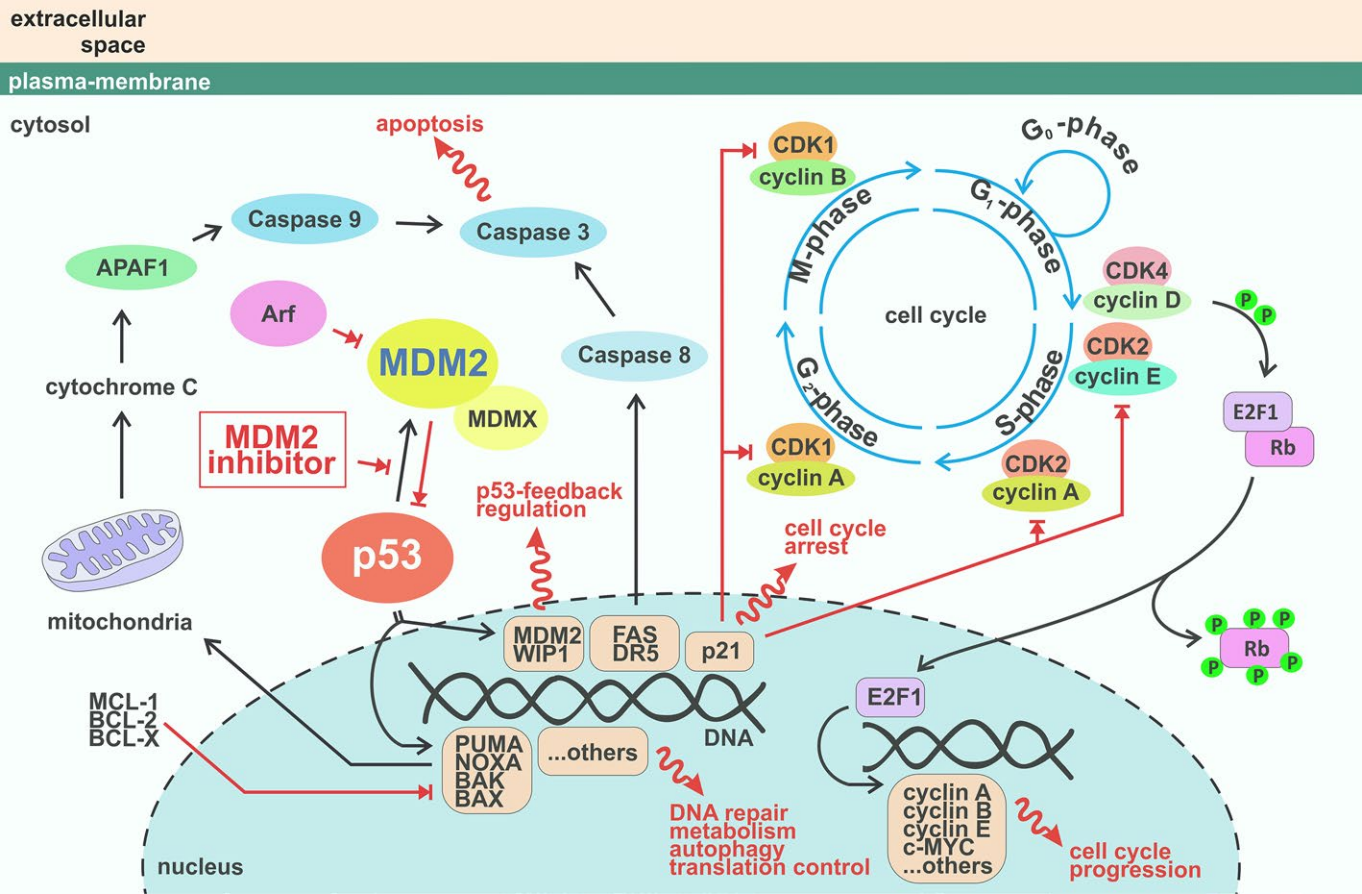
Crosstalk of cell cycle and p53 pathway. p53 activity is under the direct control of MDM2. When the MDM2-p53 interaction is interrupted via stress signals or specific MDM2 inhibitors, p53 accumulates and activates its direct transcriptional targets, resulting in protein production: p21 involved in cell cycle arrest; PUMA, NOXA, BAX, BAK involved in the intrinsic apoptotic pathway; DR4 and FAS involved in the extrinsic apoptotic pathway; MDM2, WIP1 involved in p53 feedback regulation and many others participating in DNA repair, cell metabolism, autophagy, and translational control. Cell cycle progression is controlled by p53 activity mainly via p21 protein, which associates with and inactivates CDK/cyclin complexes and blocks cell cycle progression. CDK4/6 with cyclin D/E controls the activity of RB and E2F1. When RB is hyperphosphorylated, it is released from binding to E2F1, and E2F1 then activates its transcriptional program, leading to cell cycle progression

MDM2 inhibitors alter key molecules involved in cell cycle regulation via p53-dependent regulation or crosstalk, and these may also be involved in sensitivity (Fig. 4). **Rb** (retinoblastoma protein) is a tumour suppressor involved in cell cycle progression that associates with **E2F** and represses its transcriptional activity [103]. When Rb is inactivated by phosphorylation by cyclin dependent kinase **CDK2** or **CDK4/6**, it releases E2F for cell cycle progression [104–106]. MDM2 is responsible for proteasomal degradation of hypophosphorylated Rb [107, 108] and promotes Rb translation in genotoxic conditions, resulting in G1 cell cycle arrest [109]. It was shown that Rb depletion by siRNA or inactivation via adenovirus E1A enhanced the nutlin-3-induced apoptotic response. This effect can be explained by activation of E2F transcriptional activity, with p73 induction in Rb mutant cells playing a critical role in apoptosis after nutlin-3 [110]. MDM2 upregulation by p53 activation after nutlin-3 reduced Rb phosphorylation and increased hypophosphorylated Rb in a panel of wild-type p53 cell lines, with the exception of nutlin-3 sensitive SJSA-1 cells harbouring *MDM2* amplification, where downregulation of hypophosphorylated RB was observed [111]. This downregulation is p53-dependent and occurs through induction of the cyclin-dependent kinase inhibitor **p21** (the major p53 target for inducing growth arrest, also known as p21^cip1/waf1^ or cyclin dependent kinase inhibitor 1A, *CDKN1A*), and seems to play a critical role in triggering apoptosis [111]. However, *MDM2* amplification in SJSA-1 cells cannot explain nutlin-induced Rb regulation in general, as it was not observed in other cell lines with *MDM2* amplification [111], nor is *MDM2* amplification always present in nutlin-3 sensitive cells [110]. Nutlin-3 downregulation of RB was also observed in melanoma cell lines, and E2F1 levels dictate nutlin-3 sensitivity: sensitive melanoma cells accumulated MDM2, inducing p21 and lowering E2F1 levels, whereas resistant cells accumulated MDM2 but maintained E2F1 and showed less potent upregulation of p21 [112]. Rb is often mutated and E2F1 activity increased in tumour cells, making these aspects relevant considerations for MDM2 inhibition therapy.

Cyclins are important regulators of cell cycle progression, and their crosstalk with the p53 pathway is essential. As mentioned above, Rb phosphorylation is controlled by the **cyclin D1-CDK4/CDK2** complex. Cyclin D1 is upregulated by nutlin-3, and cell lines became more sensitive by decreasing its level [113]. Cyclin D1 is commonly overexpressed in breast cancer and drives tumour growth by constitutive Rb hyperphosphorylation and E2F activation [114, 115]. In a model system of mammary cells transformed by cyclin D1/CDK activity, nutlin-3 was still able to cause p53 dependent growth arrest by repressing CDK1 and cyclin B1 [116].

Overexpression of MDM2 and CDK4 occurs in several tumour types such as liposarcoma, melanoma and osteosarcomas, and targeting both MDM2 and CDK4 is of interest in such settings, evidenced by a synergistic effect in liposarcomas [117]. This combination was subsequently evaluated in patients with locally advanced or metastatic liposarcoma (NCT02343172) (Table 1), demonstrating a manageable safety profile and favourable results compared to single-agent CDK4 inhibitor [64]. A phase II study aims to assess the efficacy and safety of this combination for patients with advanced/metastatic cancers based on their molecular alterations (NCT04116541). However, a recent study in sarcoma cell lines uncovered no synergistic effect of CDK4 and MDM2 inhibition and, on the contrary, CDK4 inhibition antagonised nutlin-3a and led to downregulation of p53 and its target genes [118]. The direct association of cyclin D1/CDK4 and p53/MDM2 seems responsible for this effect. CDK4 inhibition increased the occupancy of p53 on its target genes but diminished RNA polymerase II recruitment to these genes, resulting in decreased expression. Co-administration of MDM2 inhibitor was beneficial in melanoma cells resistant to CDK4/6 inhibition, characterised by abnormal PI3K/AKT (phosphoinositide 3-kinase/protein kinase B) signalling, and CDK4/6 inhibition upregulated cyclin D1 that sequestered p21. The addition of MDM2 inhibition caused p21 upregulation by p53, leading to CDK2 inhibition and tumour regression in patient-derived xenografts [119]. These examples illustrate the need to specifically define cellular backgrounds in which dual inhibition will be beneficial.

The involvement of cyclin **B1/CDK1** and its substrate **iASPP** (inhibitor of apoptosis stimulating protein p53) was examined in a melanoma model. Compared to normal melanocytes, iASPP, MDM2 and cyclin B1 are often overexpressed in melanoma. iASPP phosphorylated by cyclin B1/CDK1 localises to the nucleus, binds p53 and inhibits p53-mediated transcription of apoptosis related genes *PIG3*, *BAX* and *PUMA*, but not the senescence related gene *CDKN1A*. High nuclear iASPP levels were associated with metastasis and poor patient survival. Inhibiting iASPP phosphorylation by cyclin B/CDK1 inhibition, or iASPP knockdown by siRNA, enhanced the apoptotic function of p53 after nutlin-3 treatment, representing a promising strategy for melanoma [120].

#### PI3K/AKT, PTEN, mTOR and autophagy

The **PI3K/AKT** pathway is important in promoting cell cycle progression and regulates the p53 response on multiple levels (upstream/downstream). PI3K phosphorylates and activates AKT, resulting in effects such as p21 stabilisation to aid survival [121] and MDM2 localisation to the nucleus to inhibit p53 [122]. The PI3K/AKT pathway is inhibited by **PTEN** (phosphatase and tensin homologue) [123–125], an important tumour suppressor [126]. Studies of acute lymphoblastic leukaemia (ALL) revealed that nutlin-3 upregulated p53 in all wild-type p53 cells, but apoptosis was induced only in PTEN-positive cells. **Survivin** (also called BIRC5, a member of the inhibitor of apoptosis family of proteins) is also an AKT-induced protein and its silencing sensitised cells to nutlin-3. Inhibition of PI3K/AKT signalling synergised with nutlin-3 to induce apoptosis in ALL [127]. Later, it was shown that survivin is regulated by p53-dependent upregulation of p21 upon nutlin-3 treatment [128]. In contrast, ERT fibroblasts expressing E1A, RAS and hTERT remain susceptible to nutlin-3 mediated apoptosis after PTEN or p73 loss [129]. These contradictory results from different systems raise questions about their relevance for the clinic.

PI3K/AKT signalling is crucial in regulating **mTOR** (mammalian target of rapamycin) activity, and mTOR was shown to play a role in the decision between cellular senescence and quiescence after nutlin-3a treatment in HT1080 and WI-38-tert cell lines [130, 131]. Inhibiting AKT/mTOR in AML (where mTOR signalling is often constitutively activated) impedes the transcriptional activation of p53 by nutlin-3. At the same time, nutlin-3 increased mitochondrial apoptosis by p53-mediated conformational change of BAX (BCL2 associated X protein) after dual AKT/mTOR and MDM2 inhibition [132]. Combined AKT/mTOR and MDM2 inhibition caused a synergistic antiproliferative effect and accelerated apoptosis in glioblastoma multiforme cells, a cancer type with high resistance to conventional chemotherapy [133]. Nutlin-3a treatment of mantle cell lymphoma, an aggressive type of B-cell non-Hodgkin lymphoma with cyclin D1 overexpression, decreased AKT phosphorylation at Ser473, causing p53-dependent AKT/mTOR pathway inhibition mediated by **AMPK** (AMP-activated protein kinase). Interestingly, nutlin-3a led to p53 Ser15 phosphorylation but AMPK inhibition did not affect p53 Ser15 phosphorylation, indicating that this pathway occurs in the order: p53 activation—AMPK activation—mTOR inhibition [134]. AMPK activation induced by nutlin-3a was also observed in AML and led to autophagy. The authors suggested that this autophagy induction promotes apoptosis, as autophagy blockade impaired nutlin-induced apoptosis [135]. However, other studies suggest that autophagy acts against MDM2-inhibition-induced apoptosis and autophagy blockade can increase sensitivity for this treatment [136–138].

Autophagy is induced by glucose starvation and glucose levels correlate with nutlin-3a sensitivity. Resistant cells are characterised by glycolysis and elevated levels of alpha-ketoglutarate, a TCA (tricarboxylic acid) cycle metabolite, and lower levels of **OGDH** (alpha-ketoglutarate dehydrogenase) compared to responsive cells. Targeting autophagy by glucose deprivation, treatment with 2-d-glucose or the autophagy inhibitor chloroquine or bafilomycin A1 enhanced the apoptotic response to nutlin-3a, suggesting that glycolysis-related autophagy is involved in MDM2 inhibitor resistance [137, 138]. Moreover, the p53-dependent activation of AKT and transcription factor SP1 due to nutlin-3a treatment is seen in wild-type p53 MDM2 non-amplified cells only, and leads to reduced glucose metabolism and resistance to apoptosis [139]. Autophagy blockade is indeed an effective way of restoring p53-induced apoptosis in nutlin-3a treated cells, as shown by the example of autophagy induced in an **ATM** (ataxia telangiectasia mutated)-dependent manner [136]. ATM signalling is a multifunctional pathway acting against tumour growth as well as promoting it in specific conditions [140]. ATM inhibits p53-dependent cell death after MDM2 inhibition, and cells in which ATM is depleted are susceptible to killing by nutlin-3 [141]. ATM inhibition did not change p53-dependent transcription but resulted in increased mitochondrial turnover and reactive oxygen species (ROS) production [136]. Importantly, nutlin-3a can activate ATM as part of the DNA damage response [142], which promotes p53 stabilisation by phosphorylation on Ser15. Initially, it was believed that nongenotoxic activation of p53 does not lead to p53 phosphorylation [143], but several studies demonstrated that these phosphorylation events are present in various cell lines and settings and are important for p53 transcriptional function [134, 142, 144]. Moreover, activated ATM phosphorylates MDM2 on Ser395, which switches MDM2 from a negative to a positive regulator of T*P53* mRNA translation. However, in the absence of the *TP53 mRNA-*MDM2 interaction, Ser395 phospho-MDM2 has a greater capacity to ubiquitinate p53, leading to its degradation [145–147]. In addition, the interplay between MDM2-ATM-p53 and *TP53* mRNA plays a significant role in p53 stabilisation [148]. Thus, it will be important to know how these functions of ATM are orchestrated in response to MDM2 inhibitors.

#### FLT3 signalling

In AML, a malignancy with low p53 mutation frequency, broad sensitivity of wild-type p53 cells to MDM2 inhibition was observed. Interestingly, mutation of ***FLT3***, one of the most commonly mutated genes in AML leading to constitutive activation of its tyrosine kinase activity and subsequent activation of PI3K/RAS/STAT, correlates with low sensitivity to MDM2 inhibition and is a predictive marker of response [87]. Indeed, FLT3 inhibition and nutlin-3a exhibit synergy [149, 150], and there is a phase I clinical trial open for recruitment focussed on combination treatment for AML patients with mutated *FLT3* and wild-type *TP53* (NCT04496999) (Table 2).

#### MAPK/ERK signalling

Crosstalk between the p53 pathway and the **MAPK/ERK** (mitogen activated protein kinase/extracellular signal-regulated kinase) pathway plays an important role in MDM2 inhibitor response. Nutlin-3 induces phosphorylation of **MEK1/2** (dual specificity mitogen-activated protein kinase kinase 1/2) and **ERK1/2** in a p53-dependent manner but independent of p53-transcriptional activity. After nutlin-3 treatment, p53 translocates to mitochondria, leading to the generation of ROS and subsequent phosphorylation of ERK1/2 [151]. ERK1/2 signalling activates the transcription factor **ELK1**, resulting in increased expression of **BCL2A1**, an anti-apoptotic BCL2 (B-cell lymphoma-2) family protein [152]. Inhibition of ERK1/2, MEK1/2, ELK1 and BCL2A1 enhances the apoptotic response to nutlin-3, demonstrating the restrictive action of the ERK pathway on the nutlin-3 response [151, 152]. Inhibition of MAPK/ERK and MDM2 exhibits synergistic effects in AML that are dependent on upregulation of PUMA (p53 up-regulated modulator of apoptosis, alternatively BBC3) and BIM (BCL-2 interacting mediator of cell death, alternatively BCL2L11) [153]. The combination of the MDM2 inhibitor AMG-232 and the MEK1/MEK2 inhibitor trametinib was evaluated in phase Ib clinical trials (NCT02016729, NCT02110355), showing good pharmacokinetic properties and antitumour activity [50, 51]. There is one running phase I clinical study of combined MDM2 inhibitor and MEK inhibitor focused on RAS/RAF mutant and wild-type p53 colorectal carcinomas (NCT03714958).

#### p53 transcriptional-dependent and independent effects

Notwithstanding the above considerations, there is no doubt that p53 downstream signalling affects the outcome of MDM2 inhibition. The general feature of MDM2 inhibition in wild-type p53 cells is the p53-dependent upregulation of **p21** leading to cell cycle arrest [28, 37, 40, 154]. The p21 level was shown to have no decision effect on the apoptotic response to nutlin-3a in cancer cell lines [128] or in lymphoma cells in a mouse model [155]. However, p21 induction influences many response determinants, as mentioned previously for survivin, CDK4 and others [119, 128].

MDM2 inhibition also influences p53-transcription dependent regulation of genes of the intrinsic apoptotic pathway called the pro-apoptotic BH3-only members of the BCL-2 protein family, such as PUMA and NOXA (also called PMAIP1). The capacity of nutlin-3a to clear tumour cells is dependent on **PUMA** activation in a mouse model, and its loss partially protects lymphoma cells from nutlin-3a mediated killing [155]. p53 also regulates transcription of extrinsic apoptotic pathway genes such as **FAS** (also called CD95, TNFRSF6, or apoptosis antigen 1) [37, 155–157] or **DR5** (also known as TRAILR2). The p53-dependent activation of the FAS death receptor pathway by nutlin-3a plays a significant role in cell killing in cisplatin-resistant testicular carcinoma cells [158], whilst DR5 is important in breast cancer and melanoma cell lines [159, 160].

Several studies have focussed on identifying genes differentially expressed in cells sensitive to MDM2 inhibition. Differences in p53 target gene expression with and without nutlin-3 were evaluated in patient-derived B-CLL cells, showing that all wild-type p53 samples accumulated p53 after nutlin-3 treatment but only 13 out of 16 samples induced the same set of genes [161]. One study identified a 13-gene signature that predicted patient response [162], but re-evaluation in only wild-type p53 tumours revealed that this signature is not a good prediction tool [163]. Interestingly, p53 activated by DNA damage or nutlin-3a led to the same chromatin occupancy by p53 and similar chromatin changes, indicating that p53 uses the same transcriptional programme when activated by different stresses and the differences in cellular outcome are likely caused by other regulations [164]. Moreover, the same mRNA pattern was visible across cells with different sensitivity to nutlin-3, suggesting that regulation may be on the level of mRNA translation [141]. Indeed, the p53 response is regulated by post-transcriptional regulation by RNA-binding proteins and noncoding RNAs [165], and *TP53* mRNA is tightly controlled by post-transcriptional regulation in stress conditions [166]. Rizzotto et al. investigated differences in polysome-bound mRNAs (those mRNAs undergoing translation) in SJSA-1 cells undergoing apoptosis and in HCT116 cells undergoing cell cycle arrest after nutlin-3a treatment. They identified a CG-rich motif (CGPD) in differentially expressed mRNAs, which is recognised by **DHX30** (DExH-box helicase 30) and **PCBP2** (Poly(RC) binding protein 2). DHX30 expression in HCT116 cells reduced the translational efficiency of CGPD-containing mRNAs, and its depletion enhanced nutlin-3a-induced apoptosis [167].

There is an increasing body of evidence that p53 contributes to apoptosis via cytoplasmic accumulation, mitochondrial translocation and interaction with **BCL-2 protein family members** including BAX, BAK, BCL-2 and BCL-XL [168]. This transcription-independent role and the indispensability of direct interaction of p53 with mitochondrial antiapoptotic proteins for apoptosis induction after nutlin-3a treatment were reported in CLL cells [36, 169]. Surprisingly, blocking p53 transcriptional activity enhanced the mitochondrial p53 death programme and increased the overall apoptotic outcome of nutlin-3a in leukaemia and colon carcinoma cells [170].

*Bcl2l1* (BCL2-like 1) coding for BCL-XL was identified as the second most abundant factor causing resistance to the MDM2 inhibitor HDM201 in a mouse model. BCL-XL inhibition by a dual inhibitor of BCL-2/BCL-XL exerted synergistic effects in 35 of 135 cell lines tested, indicating dependence on this resistance mechanism in some cellular backgrounds [101]. These results are consistent with an AML model, where combination MDM2 and BCL-2/X-L inhibition ameliorated tumour regression [171]. A phase Ib clinical study (NCT02670044) of combination MDM2 and BCL-2 inhibitors in relapsed or refractory AML demonstrated encouraging safety and efficacy in patients who were ineligible for cytotoxic chemotherapy [172].

The balance between apoptotic versus antiapoptotic gene activation by p53 is believed to be important in cell fate decisions [173]. The effect of key antiapoptotic regulators was evaluated in nutlin-3a apoptosis resistant HCT116 cells [174]. Depletion of **MCL-1** (myeloid cell leukaemia sequence 1), **cIAP1** (cellular inhibitor of apoptosis 1) or **FLIP(L)** (Fas-associated death domain [FADD]-like interleukin-1β-converting enzyme inhibitory protein, isoform L) enhanced the response to nutlin-3a. FLIP(L) blocks caspase-8 activity and can supress p53-mediated induction of PUMA, thus counteracting apoptosis induced by nutlin-3a [174]. MCL-1 is another antiapoptotic protein from the BCL-2 family that blocks apoptosis by binding BAX and BAK. MCL-1 is upregulated in melanoma cell lines by nutlin-3a, even in p53-mutant or -null cells [160].

**NOTCH1**, a known p53 target [175], is upregulated by nutlin-3a in wild-type p53 but not mutant or null leukaemic cell lines [176]. NOTCH1 upregulation protects against apoptosis and can restrain the efficacy of the treatment. Nutlin-3a abolished osteoclastogenic events, a drawback of NOTCH signalling inhibitors, making combined treatment a promising strategy for NOTCH-dependent tumours [176].

Another p53 target gene, **WIP1** (Wild-type p53-induced phosphatase 1, also called protein phosphatase 1D), negatively regulates p53 activity. In stress conditions, WIP1 is upregulated by p53 and inhibits p53 activity by dephosphorylation of p53-Ser15 and of MDM2 and MDMX, thereby enabling cell cycle progression [177–179]. WIP1 overexpression is often found in breast cancer with wild-type p53, and dual targeting of WIP1 and MDM2 yielded synergistic effects [180]. WIP1 inhibition or depletion enhanced p53 target gene transcription in nutlin-3a treated cells, suggesting dependency of the nutlin-3a response on WIP1 levels [181].

#### Interactions with immune responses and the tumour microenvironment

p53 also influences immune responses to protect against tumour growth [3], and studies concerning the impact of wild-type p53 activation by MDM2 inhibitors on the immune response and tumour microenvironments are now emerging. Activation of the p53 response by MDM2 inhibition potentiates dendritic cell maturation, increases the level of tumour infiltrating leukocytes and induces T-cell mediated killing of tumour cells. This p53-dependent immune activation is important for targeting tumour microenvironments characterised by immunosuppressive ability [182–184]. Nutlin-3a was also shown to be crucial in activating natural killer cells via p53-dependent upregulation of ligands for **NKG2D** (NK cell receptor D, also known as killer cell lectin-like receptor K1, KLRK1), a key recognition receptor for detecting and eliminating transformed and infected cells, and **DNAM1** (DNAX accessory molecule-1; CD226) expressed on the surface of NK cells to mediate their cytotoxicity upon ligand binding [185]. Moreover, nutlin-3a induces the immune receptors **PD-L1** (programmed death-ligand 1) and **CD276** (cluster of differentiation 276, also called B7-H3) in distinct ways [186], increased CD276 is p53-dependent and is mediated via MDM2-CD276 interaction, whereas increased PD-L1 is favoured in a p53-null phenotype or in settings where p53-MDM2 interaction is disrupted by MDM2 inhibition. Upregulation of PD-L1 and CD276 by MDM2 inhibition results in antagonistic effects of this treatment by diminishing T-cell killing of cancer cells. These findings demonstrate that MDM2 inhibition may contribute to immune evasion of cancer cells. However, the immune evasion is complex and includes many other regulatory mechanisms [187]. In addition, recent results indicate that MDM2 inhibitors may sensitise tumours to T-cell mediated killing in combination with anti-PD-1 therapy, regardless of changes in PD-L1 expression [183, 188]. These reports of crosstalk between p53 and the immune response raise the following questions: What are the determinants of successful immune activation by p53 in the tumour microenvironment? And how can this be translated to improve patient-specific therapy? These questions are being addressed by clinical trials of combined MDM2 inhibitor and monoclonal antibodies targeting PD-1/PD-L1 that have started for breast cancer, advanced solid tumours, liposarcomas, metastatic melanomas, and colorectal and renal cell carcinomas (NCT03566485, NCT03611868, NCT04785196, NCT03964233, NCT02890069).

### p53-independent mechanisms of MDM2 inhibitors

Besides its main role in p53 regulation, MDM2 is also involved in DNA repair [17, 18], DNA replication [189, 190], mitochondrial dynamics [191], angiogenesis [16, 22] and gene expression [192]. As described earlier, MDM2 inhibition is generally more effective in wild-type p53 cells than those harbouring *TP53* mutations (Fig. 3). However, p53-mutant or p53-null cell lines demonstrated that MDM2 inhibition impacts the cell in p53-independent but MDM2-dependent ways. Several p53-independent responses are documented, caused mainly by disruption of MDM2 binding to other proteins, or by off-target activity of MDM2 inhibitors.

For the former, the N-terminal domain of MDM2 represents an important interaction interface for many proteins such as p73, p63, DP1, HAUSP, hTERT, NUMB and NOTCH [193], and MDM2 inhibitors that bind to this domain potentially abolish these interactions. Moreover, MDM2 exhibits structural plasticity and allosteric changes impact its functions [145, 194], implying that not only N-terminal interacting proteins are influenced by MDM2 inhibitors. One of the first pieces of evidence for a p53-independent action of nutlin-3 came from Ambrosini et al. in 2007. By comparing the effect of nutlin-3a on cell lines expressing wild-type p53, mutant p53 or lacking p53, they showed p53-independent E2F1 stabilisation, explained by inhibition of the interaction between MDM2 and E2F1. Nutlin-3a enhanced the cytotoxicity of genotoxic agents through E2F1 activation and subsequent transcription of proapoptotic p73 and NOXA [195].

The involvement of **p73** in p53-independent nutlin-3a induced cell death has been examined in several studies. TAp73α, the longest isoform of p73 and which contains the p53-like transactivation domain, is a p53 family member able to transactivate p53-responsive genes. MDM2 binds to the N-terminal region of TAp73 via its hydrophobic pocket, resulting in suppression of TAp73 transcriptional activity [196]. Nutlin-3a disrupts the interaction, leading to increased TAp73 transcriptional activity. Using siRNA against p73 or a dominant negative p73 form, the apoptotic effect of nutlin-3a in p53-mutant or p53-null cells was indeed shown to depend on p73 transcriptional activity. Moreover, possible activation via E2F1 was excluded [197]. Interestingly, nutlin-3a enhanced cytotoxicity in a doxorubicin resistant p53-mutant neuroblastoma cell line by activating both E2F1 and p73, showing its potential benefit for highly aggressive chemoresistant p53-null tumours [198].

The pro-angiogenic effect of MDM2 represents one of its oncogenic activities and is linked to the upregulation of VEGF (vascular endothelial growth factor) and **HIF-1α** (hypoxia inducible factor 1-α) in both normoxic and hypoxic conditions [199]. The crosstalk between p53 and HIF-1α is complex and they can act either in parallel or in competition, depending on cell type, type of stress, etc. This complexity is underlined by independent MDM2 interactions with HIF-1α protein and *VEGF* mRNA that influence VEGF expression [16, 199–201]. MDM2 inhibition has antiangiogenic activity through inhibiting HIF-1α activation and blocking VEGF expression [202–204]. Mechanistically, HIF-1α binds MDM2 and nutlin-3a inhibits this interaction. In p53-null cells, nutlin-3 is still able to functionally inactivate HIF-1α by dissociating MDM2 binding to the HIF-1α C-terminal transactivation domain to regulate hypoxic responses [205].

Application of an MDM2 antagonist will paradoxically lead to MDM2 upregulation due to the positive feedback loop between wild-type p53 and MDM2. This mechanism was suggested to cause partial resistance by reducing wild-type p53 activity. On the other hand, MDM2 degrades **HIPK2** (homeodomain-interacting protein kinase 2) [206], a serine-threonine kinase responsible for phosphorylation of p53 at Ser46 to enhance apoptosis [207, 208]. Thus, nutlin-3 reduces HIPK2 by MDM2-mediated degradation, resulting in mitotic arrest instead of apoptosis, and differences in HIPK2 expression or functionality therefore contribute to sensitivity to MDM2 inhibition [206].

**IGF-1R** (insulin-like growth factor type 1 receptor) is another example of a protein regulated by nutlin-3 induced upregulation of MDM2, resulting in IGF-1R degradation [209]. IGF-1R is a known MDM2 substrate and is involved in many malignancies [210–213]. MDM2 targets IGF-1R for ubiquitination through amino acids 161–400 of MDM2, residues not occupied by nutlin-3 [214]. Intriguingly, nutlin-3a triggers IGF-1R activation, a process that is independent of p53 status but dependent on interaction with MDM2, thus fine-tuning ERK activation [209] and contributing to p53-dependent nutlin-3 induced ERK signalling [151]. Furthermore, cisplatin-resistant osteosarcoma cells characterised by elevated basal activation of IGF-1R/AKT display hypersensitivity to nutlin-3a but reduced AKT-dependent autophagy flux, and inhibiting IGF-1R, AKT or autophagy flux improved the nutlin-3a response [215].

MDM2 can localise to the cytosol and mitochondria, where it can affect oxidative respiration-related proteins [216]. Nutlin-3a perturbs mitochondrial protein–protein interactions, mediated by MDM2 interaction with **DLD** (dihydrolipoamide dehydrogenase), an enzyme involved in mitochondrial metabolism [217]. Recently, the involvement of MDM2 in ROS production and mitochondrial apoptosis was demonstrated and depends on interaction with **NDFUS1** (NADH:ubiquinone oxidoreductase 75 kDa Fe–S protein 1) from the respiratory chain. Following nutlin-3a treatment, MDM2’s effect on respiration is reinforced by increased association of MDM2 and NDFUS1 [218].

As MDM2 antagonists are designed to mimic p53 residues that are involved not only in MDMD2/MDMX binding but also in many other p53-protein interactions, this type of MDM2 antagonist exerts off-target activity. Off-target binding was documented for BCL-X_L_, and BCL-2 and can contribute to p53-transcription independent mitochondrial apoptosis [219]. Another p53-independent effect of MDM2 inhibitors was explored on the level of DNA damage. MDM2 inhibition, often described as nongenotoxic, may trigger DNA damage responses in some circumstances, as documented by the formation of double-strand breaks, H2AX Ser139 phosphorylation and activation of ATM, Chk2 and BRCA1, and these events are p53-independent as they occur in p53-null cells [142, 220]. The activation of DNA damage responses was seen even after MDM2 depletion, suggesting that it is not related to MDM2 antagonism [142]. Although the mechanism responsible for MDM2-independent DNA damage by MDM2 inhibitors has not been identified, MDM2 and MDMX play roles in DNA break repair independently of p53 [18, 221]. Interestingly, the nutlin-3a induced upregulation of MDM2 in wild-type p53 and p53-null cells inhibits DNA double strand break repair [222]. Thus, it is likely that MDM2 inhibitors trigger higher mutational levels in cells.

In addition, MDM2 inhibition in p53-null or p53-mutant cells enhances the effects of other cancer treatments such as DNA damaging agents [198, 223], arsenic trioxide [224], or bortezomib [225]. Having mentioned the potential benefits of MDM2 inhibition in p53-null or -mutant cells, these are not the target group in clinical trials, which instead focus on wild-type p53 tumours.

## Acquired resistance to MDM2 inhibition

Acquired resistance constitutes one of the main obstacles for advanced and metastatic tumours. Tumours may respond well to treatment initially, but there is emergence of adapted non-responding cells by activation of oncogenes, inactivating mutations in tumour suppressors, change of the tumour microenvironment affecting drug absorption and immunosurveillance, and many other factors [226, 227]. It was thought that MDM2 inhibitors as a type of nongenotoxic target therapy would suffer less with problems of acquired resistance compared to conventional chemotherapy [228]. Unfortunately, acquired resistance represents a particular problem for MDM2 inhibitors, as even short treatment results in generation of resistant cell populations across divergent cell lines [229–231]. Resistance originating from activation of ABC transporters such as P-glycoprotein leading to efflux of chemotherapeutic agents [232] is instrumental in resistance to nutlin-3a in colorectal cancer cells [233].

The leading cause of acquired resistance of MDM2 inhibitors is attributed to the acquisition of new mutations. Adapted cells are characterised by acquiring loss of function mutations in *TP53*, mainly in its sequence coding the DNA binding domain [229, 234, 235]. Moreover, these resistant cells emerge by de novo mutations and are resistant not only to additional MDM2 inhibitor application but also to a broad spectrum of chemotherapies [234, 235]. Interestingly, the frequency of *TP53* gene mutations is much higher after MDM2 inhibition than after cytotoxic agents, indicating selection pressure for *TP53* mutated cells and suggesting that p53-independent functions of MDM2 are involved in this selection process [234, 236].

Although *TP53* mutations after prolonged MDM2 inhibition are the most frequent, other regulatory pathways are also altered and are a likely cause of acquired multidrug resistance. Whole genome sequencing and transcriptome analysis of resistant cells revealed that N-RAS (neuroblastoma RAS viral oncogene homologue), MAPK/ERK, IGFBP1 (insulin-like growth factor-binding protein 1) and NF-κB (nuclear factor-κB) are upregulated [234, 237]. Activation of these pathways would explain the emergence of resistance to cell death, but further investigation will be needed to gain a deeper understanding of the molecular processes. In a recent study, Deben et al. [238] found that resistant cells derived from non-small cell lung cancer have increased gene expression of factors that promote epithelial-mesenchymal transition. The level of the transcription factor LEF-1 (lymphoid enhancer-binding factor 1), which induces gene expression of N-cadherin, vimentin and Snail, was significantly increased in nutlin-resistant cells. Other factors upregulated in resistant clones were matrix metalloproteinases involved in tumour invasion, neoangiogenesis-related and inflammation-related proteins such as CSF1-2, IL-5, IL-13, PD-L1, PD-L2, CD73, galectin-3 and CXCL1-3 [238]. Production of these inflammatory molecules and checkpoints influences the tumour microenvironment, increases tumour cell survival and thus contributes to chemoresistance [239]. Altogether, adapted cells are characterised by loss of p53 activity due to p53 inactivating mutation, increased proliferative and invasive activity, or changes in the tumour microenvironment. Acquired resistance will compromise the effect of other chemotherapeutics that use p53 activation for clearance of tumour cells [203], or other target therapies such as MEK inhibitors [240]. Hence, it is likely that the order and combination of therapeutic approaches will be an important consideration.

The development of new MDM2 inhibitors upgraded characteristics including affinity for MDM2, cell permeability and toxicity. Nevertheless, the generation of resistant cells seems to be universal for MDM2 inhibitors as documented for RG7388 [237], MI-63 [241], HDM201 [101], idasanutlin [235] and SAR405838 [242] in both in vitro and in vivo systems. Moreover, using SAR405838, the development of secondary resistance does not depend on whether a fixed drug concentration is applied or the drug concentration is gradually increased over time [242].

Initial results from a clinical trial confirm that MDM2 inhibitor leads to a higher proportion of *TP53* mutant subclones by selection of a pre-existing cell sub-population. Interestingly, these mutant subclones decrease after treatment cessation [243]. It is not known yet whether these *TP53* mutations will compromise the effect of the treatment. Thus, monitoring adapted cells within tumour heterogeneity will be an important step in placing acquired resistance in the context of patient treatment.

## Conclusions and perspectives

**Fig. 5:**
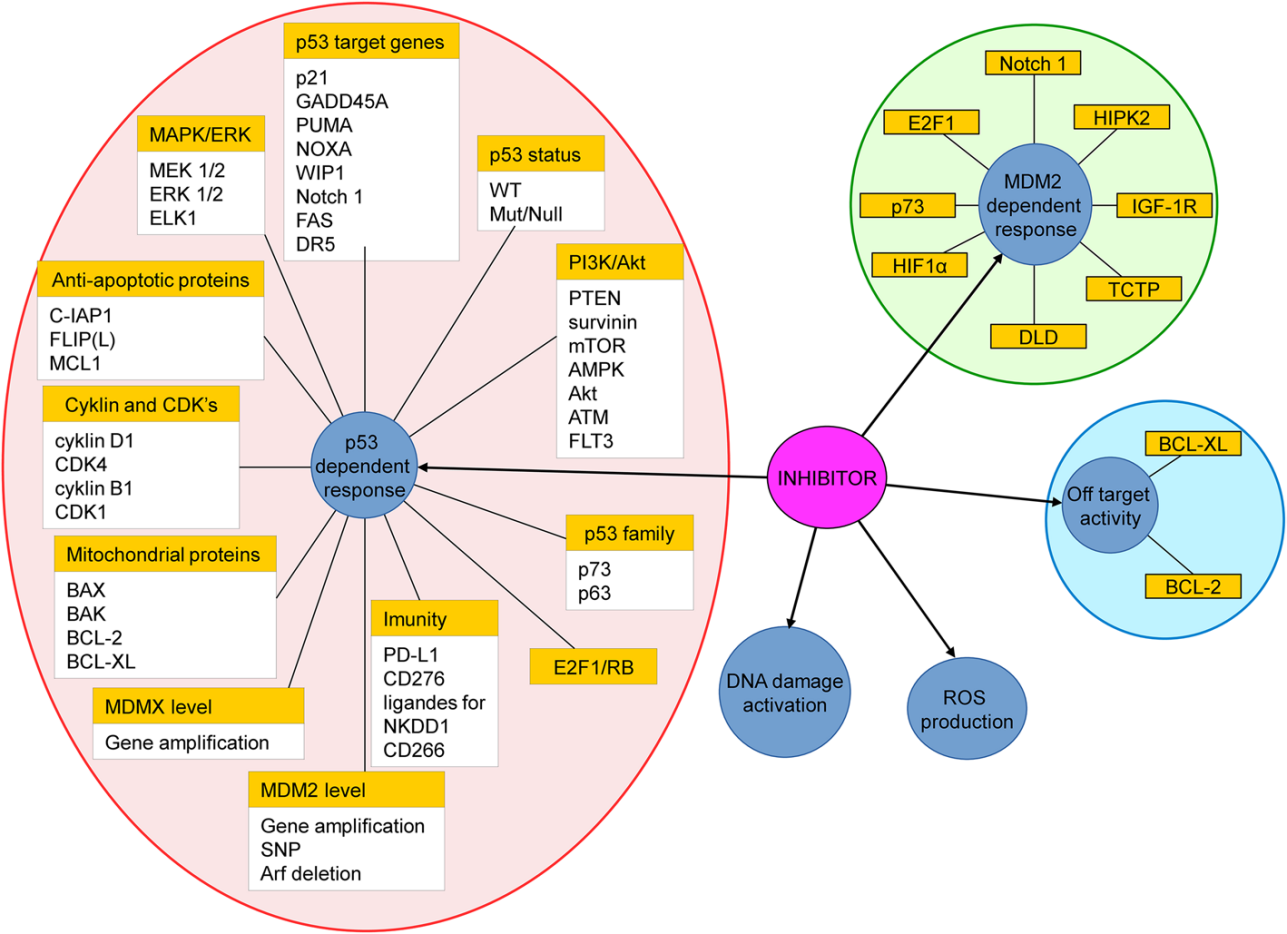
Determinants of response to MDM2 inhibitors. The overall representation of factors determining sensitivity to MDM2 inhibitors divided into MDM2-dependent determinants, p53-dependent determinants, off-targets and other determinants. The determinants are divided into related groups of proteins. All of these factors were reported in the literature to affect the sensitivity to MDM2 inhibitors designed to disrupt the p53-MDM2 interaction interface

In cancer cells bearing wild-type p53, MDM2 inhibitors brought satisfactory results regarding p53 activation. Unfortunately, the overall outcome of this activation varies greatly due to multiple factors that influence p53 pathway activation or p53-independent MDM2 functions (Fig. 5). Moreover, MDM2 inhibitor response is influenced differently by the same genetic alteration in different systems, and predicting patient response is further complicated by intratumoural heterogeneity, influence of the immune system, nutrition, etc. Efforts to find universal predictive biomarkers for MDM2 inhibition will likely fail due to this diversity. However, improved understanding of the specific pathway(s) responsible for resistance in an individual patient will aid the prediction of their specific biomarker—a panel of markers rather than a single marker will need to be assessed for optimal use of MDM2 inhibitors in personalised medicine. As an exemplar, AML commonly has wild-type p53 but has low sensitivity to MDM2 inhibitors due to *FLT3* mutation [87]. Preclinical studies showed that dual inhibition of MDM2 and FLT3 improves outcome for those patients [87, 149, 150] and a clinical trial (NCT04496999) is underway based on those findings. Similarly, whilst all of the determinants of MDM2 inhibitor responses mentioned in this review are worth further study, we do not expect that they will all play a significant role, and re-evaluation of these factors is required to identify their value for predicting response and informing treatment decisions, including the best combination therapy for the individual tumour. Unfortunately, promotion of mutant p53 subclones occurs after MDM2 inhibition, although cessation of MDM2 inhibitor leads to their regression [243]. That higher single doses of MDM2 inhibitors are more effective for activating a p53 apoptotic response [41] indicates that dose and duration of MDM2 inhibition should be reconsidered to reduce acquired resistance. Finally, results from ongoing or planned clinical trials that assess MDM2 inhibitors together with tumour-specific targeting drugs should provide useful information for identifying predictive biomarkers and for designing appropriate combination therapies in the era of personalised medicine. Although not yet a complete success, the future of MDM2 inhibitors is bright.

## Data Availability

The datasets analysed during the current study are available in the GDSC repository, [https://www.cancerrxgene.org/compound/Nutlin-3a%20(-)/1047/by-tissue?], accessed 10 March, 2021.

## Abbreviations

AKT: Protein kinase B
ALL: Acute lymphoblastic leukaemia
AML: Acute myeloid leukaemia
AMPK: AMP-activated protein kinase
ATM: Ataxia telangiectasia mutated
BAX: BCL2 associated X protein
BCL2: B-cell lymphoma-2
BIM: BCL-2 interacting mediator of cell death
CDK: Cyclin dependent kinase
CD276: Cluster of differentiation 276
cIAP1: Cellular inhibitor of apoptosis 1
CLL: Chronic lymphocytic leukaemia
CYP3A4: Cytochrome P450 3A4
DHX30: DExH-box helicase 30
DLD: Dihydrolipoamide dehydrogenase
DNAM1: DNAX accessory molecule-1
ERK: Extracellular signal-regulated kinase
FLIP(L): Fas-associated death domain [FADD]-like interleukin-1β-converting enzyme inhibitory protein, isoform L
FLT3-ITD: Fms related receptor tyrosine kinase 3 internal tandem duplication
GDSC: Genomics of drug sensitivity in cancer database
HIF-1α: Hypoxia inducible factor 1-α
HIPK2: Homeodomain-interacting protein kinase 2
iASPP: Inhibitor of apoptosis stimulating protein p53
IGFBP1: Insulin-like growth factor-binding protein 1
IGF-1R: Insulin-like growth factor type 1 receptor
LEF-1: Lymphoid enhancer-binding factor 1
MAPK: Mitogen activated protein kinase
MCL-1: Myeloid cell leukaemia sequence 1
MEK1/2: Dual specificity mitogen-activated protein kinase 1/2
mTOR: Mammalian target of rapamycin
NDFUS1: NADH:ubiquinone oxidoreductase 75 kDa Fe–S protein 1
NKG2D: NK cell receptor D
N-RAS: Neuroblastoma RAS viral oncogene homologue
NF-κB: Nuclear factor-κB
OGDH: Alpha-ketoglutarate dehydrogenase
PCBP2: Poly(RC) binding protein 2
PD-L1: Programmed death-ligand 1
PI3K: Phosphoinositide 3-kinase
PTEN: Phosphatase and tensin homologue
PUMA: P53 up-regulated modulator of apoptosis
RB: Retinoblastoma protein
ROS: Reactive oxygen species
TCA: Tricarboxylic acid cycle
VEGF: Vascular endothelial growth factor
WIP1: Wild-type p53-induced phosphatase 1
